# Host–Pathogen Cellular Communication: The Role of Dynamin, Clathrin, and Macropinocytosis in the Uptake of Giardia-Derived Extracellular Vesicles

**DOI:** 10.1021/acsinfecdis.4c00996

**Published:** 2025-03-28

**Authors:** Bruna Sabatke, Izadora V Rossi, Leticia Bonato, Sarah Fucio, Alba Cortés, Antonio Marcilla, Marcel I. Ramirez

**Affiliations:** 1 Graduate Program in Microbiology, Pathology and Parasitology, Federal University of Paraná, Curitiba, PR 81350-010, Brazil; 2 EVAHPI-Extracellular Vesicles and Host-Parasite Interactions Research Group, Carlos Chagas Institute (Fiocruz-PR), Curitiba, PR 81350-010, Brazil; 3 Graduate Program in Cell and Molecular Biology, Federal University of Paraná, Curitiba, PR 81350-010, Brazil; 4 Department of Parasitology and Cellular Biology, Faculty of Pharmacy, University of Valencia, Valencia 46010, Spain

**Keywords:** extracellular vesicles, uptake, *Giardia intestinalis*, host−parasite interaction, uptake inhibitor, endocytosis pathway

## Abstract

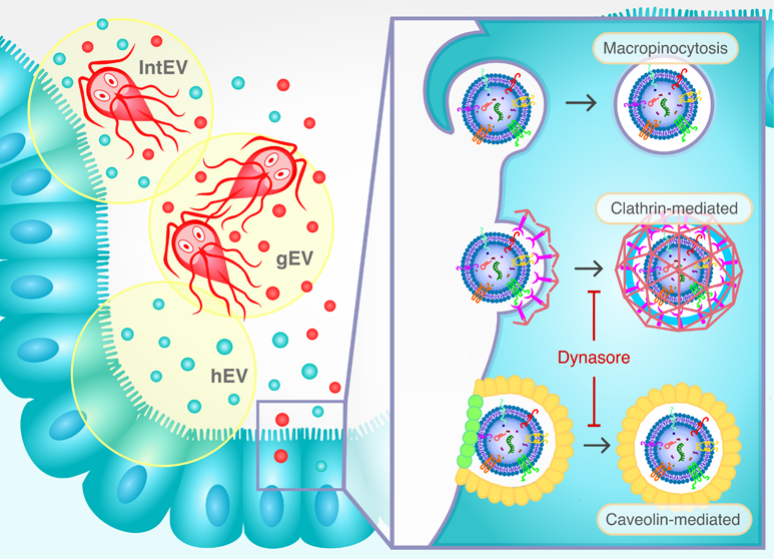

*Giardia intestinalis*, a protozoan causing giardiasis, disrupts gastrointestinal health through complex host–parasite interactions. This study explores the differential uptake mechanisms of extracellular vesicles (EVs) derived from *Giardia* (gEVs), host cells (hEVs), and the host–parasite interaction (intEVs) in intestinal Caco-2 cells. Results show that intEVs are internalized more rapidly than gEVs and hEVs, underscoring their pivotal role in pathogenesis. To delineate uptake pathways, various endocytosis inhibitors were applied, and clathrin-mediated endocytosis inhibition using monodansylcadaverine (MDC) significantly reduced intEV and gEV uptake, confirming the role of clathrin-mediated endocytosis (CME). The use of dynasore, a dynamin inhibitor, strongly reduced the internalization of all EV types, demonstrating that uptake is dynamin-dependent. In contrast, methyl-β-cyclodextrin (MβCD), which disrupts lipid rafts and caveolae-mediated pathways, had no effect on EV uptake, indicating that caveolae are not involved in this process. Furthermore, inhibition of Na^+^/H^+^ exchange and phosphoinositide 3-kinase activity, both essential for macropinocytosis, also led to a significant reduction in intEV internalization. These findings strongly support that gEVs are internalized primarily through a dynamin- and clathrin-dependent pathway, independent of caveolae and lipid rafts, but modulated by tyrosine kinase signaling and macropinocytosis. These insights into selective and comprehensive inhibition pathways offer promising therapeutic targets to mitigate giardiasis.

Intestinal parasitic infections impact about 3.5 billion people worldwide, with 450 million symptomatic cases concentrated in under-resourced areas.^1,2^ One of the most prevalent pathogens is *Giardia intestinalis* (syn. *Giardia lamblia* or *Giardia duodenalis*), a flagellated protozoan causing giardiasis, a common diarrheal illness.^3,4^ Transmission typically occurs through contaminated food, water, or fecal-oral contact. Once ingested, cysts transform into trophozoites that attach to the intestinal lining, leading to tissue damage and diarrhea.^5^ Although often asymptomatic, acute cases can persist as chronic infections, sometimes linked to drug resistance.^6^

The pathogenesis of giardiasis is a complex process that relies on the parasite’s ability to adhere to, colonize, and disrupt the intestinal epithelium, leading to gastrointestinal symptoms. Recent studies suggest that extracellular vesicles (EVs) play a key role in this process. *G. intestinalis* releases EVs throughout its life cycle, with large extracellular vesicles (LEVs) specifically involved in trophozoite adhesion to intestinal cells. Inhibiting LEV release significantly reduces parasite attachment, highlighting their importance in infection. Additionally, *Giardia*-derived EVs influence dendritic cell activation, which may contribute to immune evasion and support persistent infections.^7,8^

According to minimal information for studies of extracellular vesicles (MISEV) guidelines, EVs, comprising exosomes (30–150 nm) and microvesicles (100–1000 nm), are classified based on biogenesis, size, and molecular composition.^9^

Despite growing interest, the dynamics of EV release during parasite–host interactions remain poorly understood. Caco-2 cells, widely used as a model for intestinal epithelial cells, provide a robust system to study these interactions. These cells polarize, form tight junctions, and closely mimic the structural and functional characteristics of the human intestinal epithelium,^10^ making them ideal for investigating vesicle uptake and host–pathogen dynamics. During interactions between *G. intestinalis* and host cells, three distinct types of EVs are released: parasite-derived EVs (gEV), host-derived EVs (hEV), and hybrid EVs resulting from the host–pathogen interaction (intEV), which incorporate components from both the parasite and the host. The differential uptake of these EVs by host cells is of particular interest, as it could reveal critical mechanisms of parasitism and immune evasion that influence the course of infection. These vesicles, carrying diverse biomolecules such as proteins, nucleic acids, lipids, and sugars,^11,9^ play a central role in intercellular communication and the pathogenesis of giardiasis.

Understanding the mechanisms by which EVs are internalized by host cells is crucial for developing new therapeutic strategies. Among the approaches being investigated, inhibiting endocytic pathways has shown promise in blocking the entry of EVs, preventing their interaction with target cells. One example is Arbidol (umifenovir), an antiviral drug approved in China and Russia for treating flu, which inhibits clathrin-mediated endocytosis by preventing the fusion of the viral envelope with the host cell membrane.^12−14^ Similarly, considering that endocytosis is the primary route for EV internalization, understanding the involved pathways could pave the way for innovative therapeutic interventions.^15^ Among the various endocytic routes, dynamin-dependent endocytosis stands out. Dynamin, a GTPase essential for vesicular trafficking, regulates the scission of newly formed vesicles from the plasma membrane, a crucial step in clathrin-mediated endocytosis and other cellular uptake processes. In addition, EVs can be internalized through clathrin-independent mechanisms, including caveolin-mediated endocytosis, lipid raft-mediated uptake, macropinocytosis, and phagocytosis.^16,17^ Notably, dynamin inhibitors, such as dynasore, have shown significant efficacy in blocking EV internalization, highlighting the therapeutic potential of targeting these pathways to interfere with EV-mediated intercellular communication.^18^

In this study, we investigate the differential uptake mechanisms of EV types during the interaction between *G. intestinalis* and intestinal cells. By elucidating these pathways and evaluating the effectiveness of specific endocytosis inhibitors, we aim to identify innovative therapeutic strategies that could disrupt host–parasite interactions, thereby reducing the burden of giardiasis.

## Results

### Characterization of EVs Released during Parasite–Host Cell Interaction

**Figure 1:**
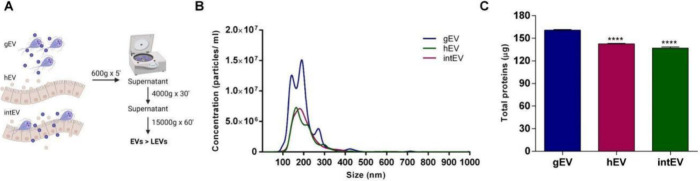
Characterization of extracellular vesicles isolated during host–parasite interaction. (A) Schematic representation of the differential centrifugation process used to isolate different types of EVs: *G. intestinalis*-derived EVs (gEVs), host-derived EVs (hEVs), and EVs resulting from host–parasite interaction (intEVs). (B) Particle size distribution analyzed by nanoparticle tracking analysis (NTA). (C) Protein concentration of gEVs, hEVs, and intEVs measured using a protein-based assay.

During the interaction between*G. intestinalis* and Caco-2 cells, three distinct types of extracellular vesicles (EVs) can be identified: vesicles originating from *G. intestinalis* (gEV), vesicles released by Caco-2 host cells (hEV), and interaction vesicles formed during the parasite–host interaction (intEV), which may incorporate membrane components from both the parasite and host cells (Figure 1A). Initially, we characterized gEVs, hEVs, and intEVs using nanoparticle tracking analysis (NTA) and protein quantification. The NTA results revealed that gEVs are the most abundantly produced EVs, with a similar size distribution across the different EV types, ranging between 150 and 300 nm, with a peak at approximately 200 nm (Figure 1B). Intriguingly, protein quantification did not reveal significant differences between the EVs, despite the higher number of gEVs, suggesting possible differences in vesicle cargos (Figure 1C).

### Tracking EV Internalization in Human Intestinal Cells

**Figure 2:**
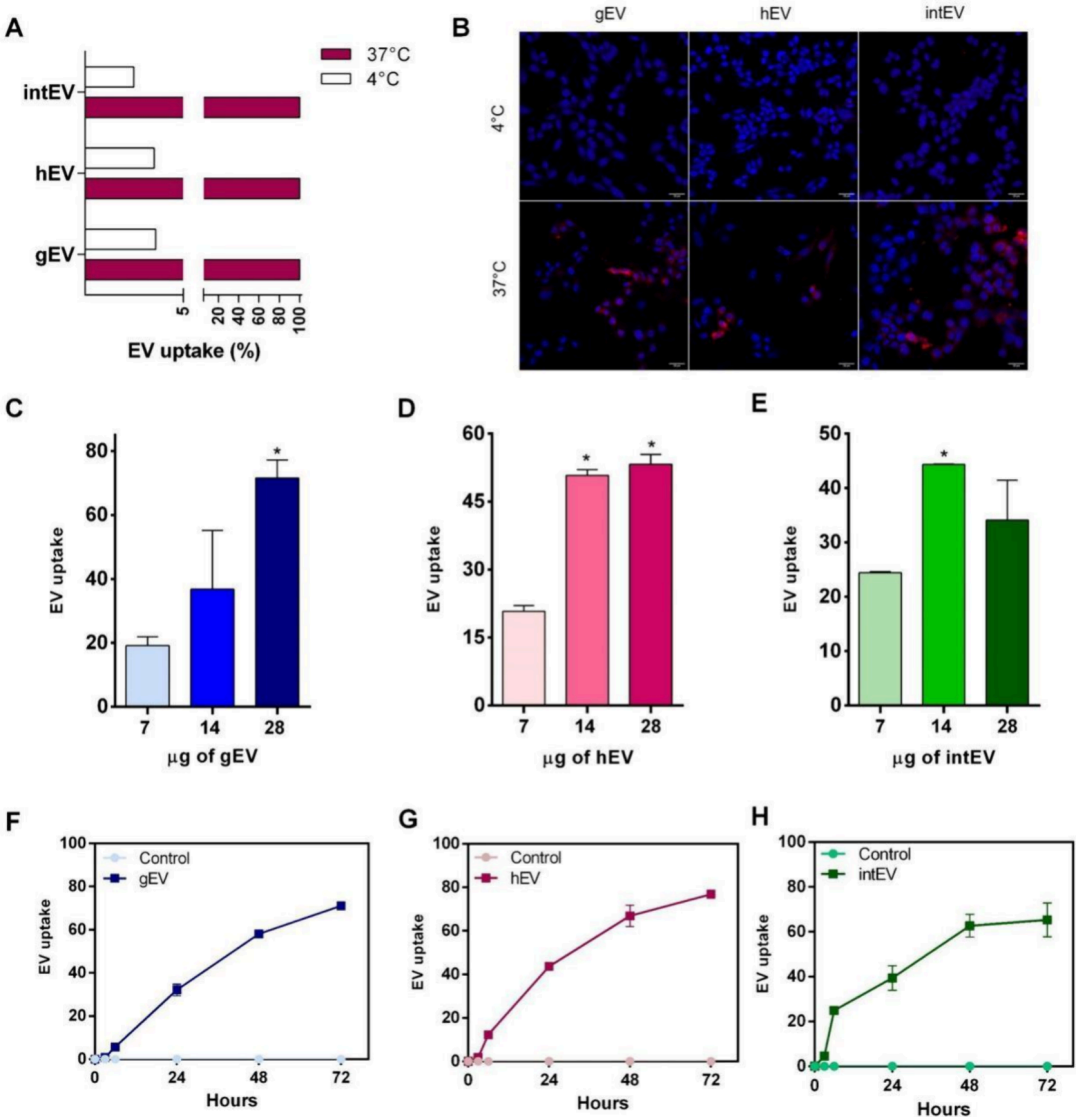
Tracking of extracellular vesicle internalization into Caco-2 cells. (A) Flow cytometric analysis of EV uptake at 37 °C (pink bars) and 4 °C (white bars). (B) Confocal microscopy images of Caco-2 cells incubated with PKH26-labeled EVs (red) at 4 and 37 °C; nuclei were stained with DAPI (blue). (C–E) Dose-dependent EV uptake into Caco-2 cells after 6 h for gEVs (C), hEVs (D), and intEVs (E). (F–H) Time-dependent kinetics of EV internalization into Caco-2 cells for gEVs (F), hEVs (G), and intEVs (H) over a 72 h period.

To explore the internalization of EVs by human intestinal Caco-2 cells, we labeled purified gEVs, hEVs, and intEVs with the fluorescent dye PKH26. A key observation was that EV internalization was not completely halted at 4 °C. However, a reduction in uptake of approximately 95% was observed by cytometry for all EVs, indicating that the process is mediated by active, energy-dependent endocytic mechanisms (Figure 2A). Confocal microscopy supports the findings, showing that EV uptake occurs exclusively at 37 °C (Figure 2B). Additionally, our experiments revealed that EV internalization exhibited both dose-dependent trends (Figure 2C,D) and time-dependent kinetics (Figure 2E–G). Notably, EVs displayed distinct internalization patterns, particularly at the initial stages, with intEVs being internalized more rapidly than gEVs and hEVs within the first 6 h, although this effect normalized over time.

### Uptake of Extracellular Vesicles by Intestinal Cells

**Figure 3:**
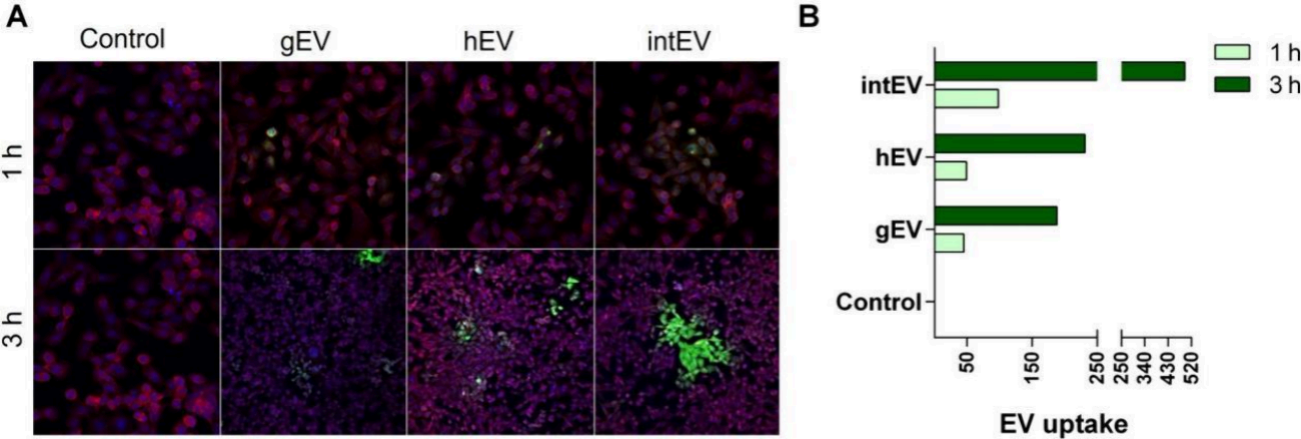
EVs from the parasite–host cell interaction (intEV) are rapidly acquired by intestinal cells. (A) Confocal microscopy images comparing the internalization of *G. intestinalis*-derived EVs (gEVs), host–derived EVs (hEVs), and EVs resulting from host–parasite interaction (intEVs) in Caco-2 cells after 1 and 3 h of incubation. The control shows no EV signal. Internalized EVs are highlighted in green (PKH67), while cell nuclei are shown in blue (DAPI), and the cytoskeleton in red (phalloidin). (B) Quantification of EV uptake based on fluorescence intensity after 1 and 3 h. Scale bar = 10 μm.

To verify the faster internalization kinetics of intEVs and to qualitatively assess their intracellular localization within Caco-2 cells, we utilized confocal microscopy. This technique allowed us to observe not only the precise intracellular positioning of the vesicles but also the total cargo internalized by the cells. By comparing the three EV types at 1 and 3 h, we initially noted a uniform uptake across all types within the first hour. However, by the 3 h mark, confocal imaging revealed a significant increase in the internalization of intEVs by Caco-2 cells, markedly higher than the uptake of hEVs and gEVs (Figure 3). These observations, supported by the detailed imaging provided by confocal microscopy, align with the quantitative data obtained from flow cytometry analysis, underscoring the distinct internalization dynamics of intEVs.

### Dynamin, Clathrin, and Macropinocytosis-Dependent Uptake Mechanisms of *Giardia* Extracellular Vesicles in Host Cells

**Figure 4:**
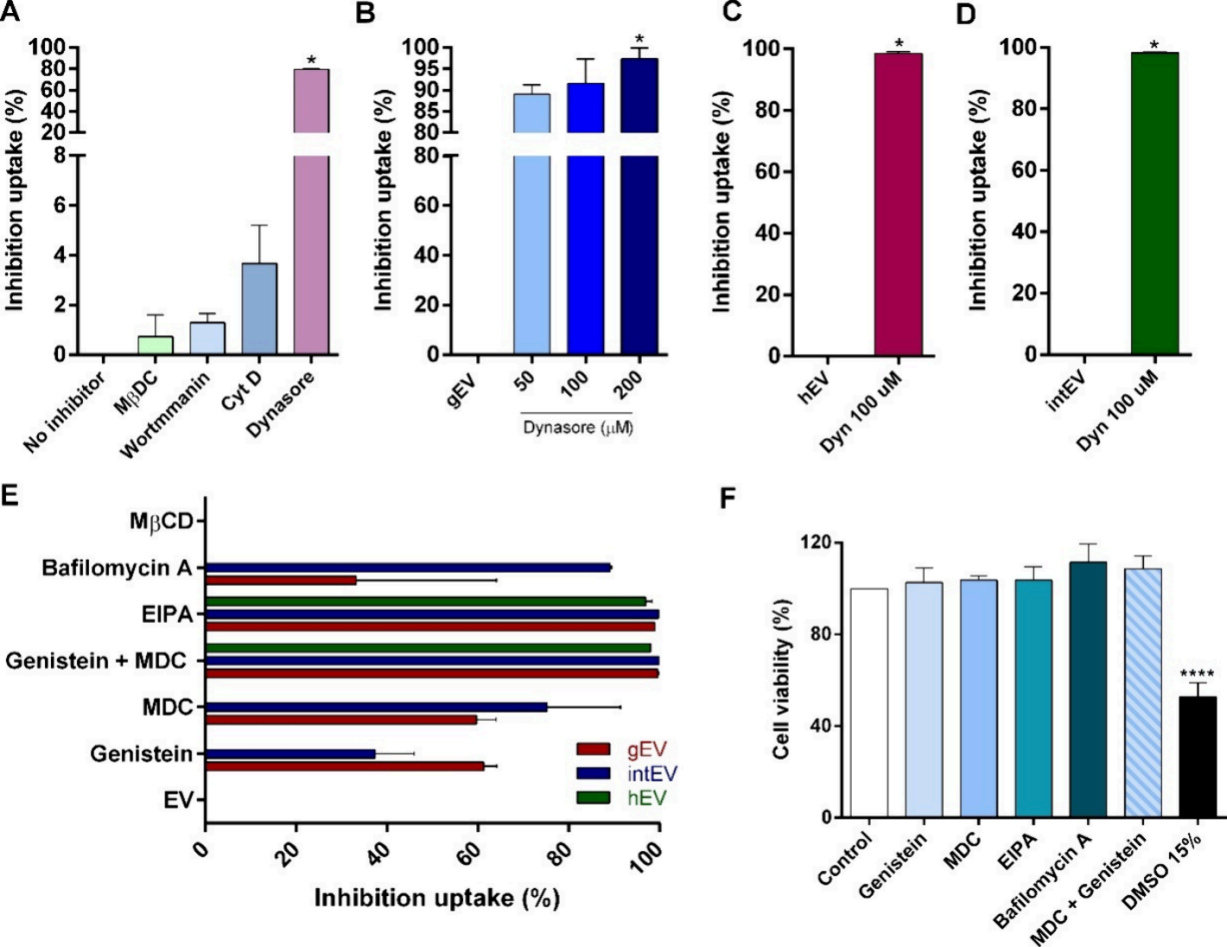
Uptake of EVs is dynamin and clathrin-dependent on Caco-2 cells. (A) Flow cytometry analysis showing inhibition of *G. intestinalis*-derived EV (gEV) uptake in the presence of different pathway inhibitors, including methyl-β-cyclodextrin (MβCD), wortmannin, cytochalasin D, dynasore, and control (Ctrl, without an inhibitor). (B) Dose-dependent inhibition of gEV uptake by dynasore at concentrations of 50, 100, and 200 μM. (C, D) Inhibition of host-derived EV (hEV) and interaction-derived EV (intEV) uptake by dynasore at 100 μM. (E) Percentage inhibition of gEV, hEV, and intEV uptake by various endocytosis inhibitors, including bafilomycin A1, EIPA, genistein, MDC, MβCD, and dynasore. (F) Cell viability assay with endocytosis inhibitors, bafilomycin A1, EIPA, genistein, MDC dynasore, and positive control (DMSO 15%).

To elucidate the specific endocytic pathways involved in EV internalization, we performed experiments using Caco-2 cells pretreated with inhibitors targeting various pathways. Dynasore, an inhibitor of clathrin and caveolin-mediated endocytosis, proved highly effective, inhibiting approximately 90% of gEV uptake, as demonstrated by flow cytometric analysis. In contrast, other inhibitors, such as cytochalasin D (an actin polymerization inhibitor), wortmannin (a phagocytosis inhibitor), and methyl-β-cyclodextrin (MβCD, a lipid raft inhibitor), showed no significant inhibitory activity (Figure 4A). The inhibition of gEV uptake by dynasore was dose-dependent, suggesting a critical role for clathrin- and caveolin-mediated pathways in EV internalization (Figure 4B). Notably, dynasore almost completely blocked the uptake of both hEVs and intEVs (Figure 4C,D). We also employed a range of inhibitors targeting both dynamin-dependent and dynamin-independent endocytosis. Dynamin-dependent pathways, which include clathrin-mediated and caveolin-mediated endocytosis, were inhibited using genistein and monodansylcadaverine (MDC), allowing us to assess the role of clathrin and caveolin in EV uptake. Additionally, we used bafilomycin A1 and EIPA to explore dynamin-independent pathways, such as macropinocytosis and endolysosomal trafficking. Figure 4E indicates that intEV uptake was significantly more inhibited, especially by bafilomycin A, MDC, and the genistein/MDC treatment. Bafilomycin A reduced intEV uptake by ∼90%, suggesting that these vesicles rely heavily on acid-sensitive pathways. EIPA uniformly inhibits uptake across all vesicle types, implicating Na^+^/H^+^ exchange in their internalization. The genistein and MDC combination had a synergistic effect, particularly on intEVs, suggesting multiple endocytic pathways. Individually, MDC reduced intEVs, while genistein affected gEVs, indicating that both intEVs and gEVs rely more on clathrin- and caveolin-mediated pathways than hEVs. In contrast, treatment with MβCD, which disrupts lipid rafts and caveolae-mediated pathways, did not significantly reduce the uptake of any EV type, reinforcing that caveolae are not involved in gEV internalization.

To ensure that the observed uptake inhibition was not due to cytotoxic effects, the cell viability of Caco-2 cells in the presence of these uptake inhibitors was also assessed (Figure 4F). Genistein, MDC, EIPA, bafilomycin A, and the genistein-MDC combination maintained cell viability above 80%, indicating minimal cytotoxicity and confirming their suitability for uptake studies without compromising cellular health, being not necessary to normalize the number of cells or the percentage of uptake. In contrast, 15% DMSO significantly reduced viability, serving as a positive control for cytotoxicity. These results validate that the observed inhibition effects on uptake are due to specific interference with vesicle internalization mechanisms and not for cell death. We also conducted an experiment to evaluate the effect of vesicles on cell proliferation. The results demonstrated that intEVs significantly increased the proliferation of Caco-2 cells at 48 h, compared with hEVs and gEVs, which did not show notable effects on cell growth (data not shown).

## Discussion

### Different Types of Extracellular Vesicles Are Produced during Parasite–Host Cell Interaction

EVs released by protozoan parasites have been documented across various species, including *Trichomonas vaginalis*,^19^*Leishmania*,^20,21^*Toxoplasma gondii*,^22,23^ and *Plasmodium*,^24,25^ among others. In the case of *Giardia*, our findings indicate that EVs generated during parasite–host cell interactions exhibit an average size of 150–300 nm, consistent with previous research.^8^ Determining the extent to which the membranes of the parasite or the host cells contribute to the formation of interaction vesicles is challenging. However, a study by Ramirez^26^ revealed that EVs derived from the interaction between metacyclic forms of *Trypanosoma cruzi* and THP-1 cells contain proteins from both the pathogen and the host, suggesting potential membrane fusion during EV release. Understanding whether these mixed vesicles play a differential role during parasite–host cell interactions is of our particular interest, even more so when only few studies have delineated the differences in EV origin.^27^ Following the recommendations of MISEV, we conducted at least two experiments using NTA (nanoparticle tracking analysis) combined with focal immunofluorescence and flow cytometry to dynamically demonstrate the structure of extracellular vesicles internalized in eukaryotic cells.

### Dynamics of Extracellular Vesicle Uptake during Parasite–Host Cell Interaction

Understanding the mechanisms by which EVs are internalized by host cells is crucial for unraveling the infection processes and pathogenesis established by *G. intestinalis*. Our findings indicate that the internalization of EVs occurs predominantly via endocytosis, consistent with a previous study.^16^ The significant reduction of EV uptake at 4 °C, which we observed, supports the notion that this process is energy-dependent, corroborating results from several studies that show a similar trend.^28,15,29^

Our flow cytometry results reveal distinct internalization patterns for the three types of EVs, with intEV being internalized more rapidly than the other two types of EVs within the first 6 h. This rapid uptake suggests that intEVs possess specific surface components or signaling molecules that enhance their recognition and interaction with Caco-2 cells, which warrants further investigation.^30,31^ This finding aligns with previous research indicating that EV uptake is influenced by time–dose dependence, as we also observed in our experiments.^32,33,15^

The confocal microscopy data offer important insights into the internalization dynamics of different EV types. The uniform uptake observed within the first hour, followed by a notable increase in intEV uptake by the 3 h mark, highlights the complex interactions between these vesicles and host cells. The distinct internalization profiles of gEVs, hEVs, and intEVs suggest that each type may utilize specific mechanisms for uptake. For instance, NK cells and epithelial cell lines exhibit distinct preferences for exosome uptake. NK cells show a greater capacity to internalize exosomes originating from themselves and bone marrow-derived cell lines, whereas epithelial cell lines demonstrate a preference for absorbing exosomes derived from other epithelial cells.^34^

### Participation of EVs in Parasite–Host Cell Communication Is Dependent on Endocytosis and Can Be Blocked by Inhibitors

To investigate the pathways involved in extracellular EV uptake, we employed specific inhibitors targeting endocytosis and macropinocytosis. Our results indicated that clathrin-, dynamin-, and macropinocytosis-mediated mechanisms are crucial in this process, consistent with prior studies highlighting these endocytic pathways as fundamental for EV uptake.^16^ These findings underscore their significance in cellular communication and host–pathogen interactions.^35,36^

Our data strongly suggest that the internalization gEV follows a clathrin- and dynamin-dependent pathway, independent of caveolae and lipid rafts but modulated by tyrosine kinase activity and macropinocytosis. This conclusion is supported by the substantial inhibition of EV uptake by dynasore, a dynamin inhibitor, which reduced the internalization of gEV, hEV, and intEV by approximately 90%. Dynasore functions as a GTPase inhibitor, rapidly and reversibly blocking dynamin activity, thereby preventing endocytosis. This observation is in line with previous studies. For instance, Guidi^37^ found that dynasore significantly reduced the internalization of bacterial outer membrane vesicles (OMVs) and the subsequent DNA damage caused by toxins, suggesting the involvement of dynamin-dependent endocytosis in the uptake of OMVs loaded with typhoid toxin. Similarly, Toribio^38^ reported a significant decrease in EV uptake upon dynasore treatment, inhibiting up to 80% of *Bacillus thuringiensis* (Bt) OMV internalization in intestinal epithelial cells. These studies highlight the potential of targeting dynamin-dependent pathways to disrupt parasite–host interactions and inhibit pathogenic EV communication at the cellular level. Additionally, Joshi et al.^39^ demonstrated that neuronal cells internalize EVs via a dynamin-dependent pathway, with dynasore treatment leading to a significant reduction in uptake. Our findings further support the essential role of dynamin in the internalization of both parasite-derived and host–parasite interaction vesicles.

To further elucidate the endocytic mechanisms involved in EV uptake by Caco-2 cells, confocal microscopy played a pivotal role. This imaging technique provided high-resolution visualization of the EV internalization process, offering critical insights into specific endocytic pathways and enabling precise localization of EVs within the cellular environment.^30,16^

The involvement of clathrin- and caveolin-mediated pathways in EV endocytosis has been highlighted in several studies. Rai and Johnson^35^ demonstrated that EV internalization from *Trichomonas vaginalis* is caveolin-dependent and regulated by host cell caveolin-1. Similarly, Bajic et al.^36^ showed that EVs from *Lactiplantibacillus plantarum* BGAN8 are internalized by HT29 cells via clathrin-mediated endocytosis (CME) without requiring cholesterol-enriched lipid rafts. The role of CME in EV uptake was further supported by inhibitors such as chlorpromazine and dynasore.

To refine our understanding of these uptake mechanisms, specific inhibitors were used. Genistein (a tyrosine kinase inhibitor) and MDC (a clathrin-mediated endocytosis inhibitor) significantly reduced the uptake of intEV and gEV, though with differing sensitivities. gEV uptake was more strongly affected by genistein, suggesting a preferential reliance on tyrosine kinase activity, whereas intEV uptake was more profoundly inhibited by MDC, confirming a clathrin-dependent route. These results align with previous studies showing clathrin-mediated EV uptake in other models. Escrevente et al.^40^ demonstrated that ovarian cancer exosomes are internalized via CME, with clathrin knockdown or pharmacological inhibition significantly blocking their uptake.

Notably, the combination of genistein and MDC produced a synergistic effect, further supporting the involvement of dynamin in both pathways and indicating a degree of interaction between clathrin- and caveolin-mediated endocytosis. However, treatment with MβCD, which disrupts lipid rafts and caveolae-mediated pathways, did not significantly reduce the uptake of any EV type, suggesting that caveolae are not involved in gEV internalization. Additionally, tyrosine kinase signaling appears to modulate these pathways, as genistein alone significantly reduced gEV uptake, consistent with Sorkin and Goh’s^41^ findings that tyrosine kinase activity regulates CME during receptor internalization. This suggests that tyrosine kinase signaling may influence EV uptake efficiency and, consequently, impact host–pathogen communication.

Furthermore, using EIPA (an Na^+^/H^+^ exchanger inhibitor), we confirmed a key role for macropinocytosis in EV entry, as indicated by a high level of inhibition (above 90%) across all EV types (gEV, hEV, and intEV). This suggests that macropinocytosis, influenced by ion exchange and intracellular acidification, is a critical pathway for EV uptake, regardless of vesicle origin. Costa Verdera^42^ proposed that EV internalization primarily involves clathrin-independent endocytosis and macropinocytosis, dependent on cholesterol, tyrosine kinase, Na^+^/H^+^ exchange, and phosphoinositide 3-kinase activity, which are vital for macropinocytosis.

Lastly, bafilomycin A1, an inhibitor of vacuolar H^+^-ATPase, inhibited intEV uptake by ∼90% with a moderate effect on gEV, indicating that intEVs rely on acid-sensitive pathways like acidified endosomes. This adaptation likely enables intEVs to exploit pH-regulated mechanisms for effective internalization, potentially reflecting a strategic adaptation by parasites.

The observed difference indicates that intEVs may play a crucial role in shaping the proliferation of intestinal epithelial cells, potentially aiding in the maintenance of intestinal homeostasis during pathogen interactions. This positive effect on cell growth supports the idea that intEVs are significant in host–pathogen dynamics, influencing not only how quickly cells multiply but also helping to preserve the integrity of the intestinal barrier over time.

In summary, EV internalization by host cells is an active, energy-dependent process clathrin- and dynamin-dependent pathway, independent of caveolae and lipid rafts, but modulated by tyrosine kinase activity and macropinocytosis. The distinct internalization profiles of gEVs, hEVs, and intEVs suggest that these vesicles are tailored to exploit specific pathways within the host cells, thereby enhancing the parasite’s ability to thrive within the host environment. Understanding these pathways not only advances our knowledge of cellular communication and pathogen–host interactions but also opens avenues for developing targeted therapeutic strategies to modulate EV-mediated processes in diseases such as giardiasis.

## Methods

### *G. intestinalis* Isolates and Caco-2 Cell Culture

The *G. intestinalis* WB isolate (ATCC 50803) was cultured in a TYI-S-33 medium,^40^ supplemented with 10% heat-inactivated adult bovine serum (ABS) and 1% penicillin/streptomycin (1000 U/mL, Gibco), at 37 °C under microaerophilic conditions. Cultures were maintained in 13 mL polystyrene tubes (BD Biosciences) until reaching confluency (1 × 10^6^ cells/mL), after which they were subcultured every 72 h. Human colorectal adenocarcinoma Caco-2 cells (ATCC CRL-2102) were cultured in an RPMI-1640 medium, enriched with 10% fetal bovine serum (FBS) and 1% penicillin/streptomycin (1000 U/mL, Gibco). The cells were incubated at 37 °C in a 5% CO_2_ atmosphere until a confluent monolayer was attained.

### Isolation of Extracellular Vesicles (EVs)

EVs were isolated from *Giardia* (gEV) and Caco-2 cells (hEV) and from the parasite–host interaction (intEV) as follows: For gEVs, parasites were detached from confluent cultures by chilling on ice for 15 min, followed by two centrifugations at 600*g* for 5 min each. After counting with a hemocytometer and diluting to 1 × 10^6^ cells per sample, the suspensions were transferred to 1.5 mL microtubes and incubated with 1 mM CaCl_2_ for 1 h at 37 °C with a serum-free TYI-S-33 medium (an option from MISEV to EV depleted serum to minimize contaminant EVs), supernatants were then subjected to centrifugation at 600*g* for 5 min to remove the parasites, followed by centrifugation at 4000*g* for 30 min to eliminate cellular debris and finally at 15,000*g* for 1 h to isolate large extracellular vesicles (LEVs) as defined before by Gavinho et al.^8^

For hEV isolation, Caco-2 cells were seeded at a density of 1 × 10^5^ cells per well and incubated for 24 h until confluency was achieved. After the culture medium was removed and the cells were washed with a serum-free medium, a medium containing 1 mM calcium was added, followed by 1 h of incubation. The EV isolation procedure was identical to that employed for the parasite-derived EVs. To obtain intEVs, Caco-2 cells were coincubated with parasites at a ratio of 1:10 for 1 h in the presence of 1 mM calcium, following the same isolation protocol. Once collected, all EVs were resuspended in phosphate-buffered saline (PBS) and stored at 4 °C.

### EV Quantification and Characterization

The protein concentration in EVs was determined using the Micro BCA protein assay kit (Thermo Fisher Scientific, cat. no. 23227), following the manufacturer’s protocol. For EV lysis, heat shock was used. For the colorimetric reaction, 25 μL of the EV sample, in a 1:5 dilution, was mixed and incubated with 200 μL of the Micro BCA reagent. A standard curve was made by diluting bovine serum albumin (BSA) in PBS 1× from 0 to 2000 μg/mL. The plate was incubated at 37 °C for 30 min, and absorbance was measured at 562 nm using a BioTek Synergy H1 microplate reader.

Nanoparticle tracking analysis (NTA) was performed by diluting each sample 1:50 in PBS (1×) and analyzing them using an NS300 Nanosight (Malvern, U.K.), with readings taken in triplicate during 60 s videos at 10 frames per second at room temperature. The parameters were as follows: camera shutter, 1492; camera gain, 512; detection threshold, 10.

### EV Staining

For uptake assays, EVs were stained with the lipophilic dye PKH26 (Sigma-Aldrich). Two microliters of the dye was diluted in 1 mL of diluent C, and both EV populations were diluted 1:40 in diluent C. The labeling process was conducted for 15 min at room temperature in the dark. The reaction was halted by adding 1 mL of fetal bovine serum, followed by washing with PBS (1×) and centrifugation at 15,000*g* for 1 h.

### Inhibitor Treatments

The concentrations of inhibitors used were based on the study established.^41^ Caco-2 cells were preincubated with the following inhibitors: 80–200 μM dynasore (clathrin and caveolin inhibitor), 10 μM methyl-β-cyclodextrin (MBCD, lipid raft/caveolae inhibitor), 1 μM wortmannin (phagocytosis inhibitor), 2.5 μM cytochalasin D (actin polymerization inhibitor), 200 μM genistein (caveolin inhibitor), 200 nM bafilomycin A (inhibitor of vacuolar H^+^-ATPase), 100 μM monodansylcadaverine (MDC) (clathrin inhibitor), and 100 μM ethyl-isopropyl amiloride (EIPA) (a Na^+^/H^+^ exchanger inhibitor/macropinocytosis inhibitor) for 30 min prior to EV addition as described in other protocols, and the inhibitors were maintained in the medium throughout the experiments.

### Uptake Assay

For flow cytometry, Caco-2 cells were seeded at a density of 1 × 10^5^ cells per well in 24-well plates. After 24 h, cells were washed and incubated with RPMI containing 10% FBS, with or without inhibitors, for 30 min as a pretreatment. The cells were then incubated with PKH26-labeled EVs for 6 h in the presence of inhibitors. After incubation, the cells were washed with PBS, trypsinized for 5 min at 37 °C, neutralized with 10% FBS, and fixed in 2% paraformaldehyde (PFA) in PBS. Samples were analyzed using a FACSCanto flow cytometer (BD Biosciences). As a negative control, PKH26 was included with the cells (not shown).

For fluorescence microscopy, Caco-2 cells were seeded at a density of 1× 10^5^ cells per well on coverslips. After 24 h, cells were incubated with PKH67-labeled EVs for 1 and 3 h. Following incubation, the cells were washed with PBS and fixed with 2% PFA at room temperature for 20 min. Intracellular membranes were visualized using Alexa 647-phalloidin, and nuclei were stained with DAPI (1:1000) at room temperature. Slides were stored at 4 °C until analysis. Cells were mounted with 20% glycerol, and internalized EVs were detected using confocal microscopy (Nikon A1R HD Multiphoton Confocal, Nikon, Tokyo, Japan). Images were processed using FIJI ImageJ software (version 1.56).

### Resazurin Cell Viability Assay with Endocytosis Inhibitors

To evaluate the viability of Caco-2 cells in the presence of uptake inhibitors (EIPA, genistein, MDC, and bafilomycin A), a resazurin assay was conducted. Caco-2 cells were seeded in 96-well plates at a density of 2 × 10^4^ cells per well and incubated for 24 h. After the serum-containing medium was removed, cells were exposed to a medium containing each inhibitor at the same concentrations used in the 3 h uptake assay, followed by incubation for an additional 3 h. Control wells with the medium alone were included for background subtraction and gain adjustment. Subsequently, 20 μL of resazurin solution (0.15 mg/mL in PBS, pH 7.4) was added to each well, and the plate was incubated for 2 h at 37 °C. Fluorescence was measured using a BioTek Synergy H1 microplate reader with excitation and emission filters set at 560 and 590 nm, respectively.

### Statistical Analysis

All quantitative data were analyzed using GraphPad Prism 6.0, with experiments conducted in triplicate. Data are presented as the mean ± standard error of the mean (SEM). Statistical significance was determined using an unpaired *t* test or one-way analysis of variance (ANOVA) with Tukey’s or Dunnett’s post hoc multiple comparison tests, as appropriate. A *p*-value of <0.05 was considered statistically significant.
